# Rac1 is a novel therapeutic target in mantle cell lymphoma

**DOI:** 10.1038/s41408-018-0052-0

**Published:** 2018-02-12

**Authors:** Tian Tian, Chengfeng Bi, Ashley L. Hein, Xuan Zhang, Cheng Wang, Songfei Shen, Ji Yuan, Timothy C. Greiner, Charles Enke, Julie Vose, Ying Yan, Kai Fu

**Affiliations:** 1 Department of Pathology and Microbiology, University of Nebraska Medical Center, Omaha, NE USA; 2 Department of Radiation Oncology, University of Nebraska Medical Center, Omaha, NE USA; 3 Department of Oncology, Fuzhou Cancer Hospital, Fujian, China; 4 Department of Hematology Oncology, University of Nebraska Medical Center, Omaha, NE USA

Mantle cell lymphoma (MCL) is an aggressive B-cell lymphoma, comprising 6–8% of human B-cell non-Hodgkin lymphomas^1^. R-CHOP, which combines rituximab with cyclophosphamide, hydroxydaunorubicin (Doxorubicin/Adriamycin), oncovin (Vincristine) and prednisone, is the most common regimen employed for treating MCL. Nevertheless, most patients are destined to relapse after initial therapy^2^, highlighting the urgent need for new therapeutic strategies. Here, we explore a member of Rac family of small guanosine triphosphatases (GTPase), Rac1, as novel target for MCL treatment.

Rac family proteins cycle between active GTP-bound and inactive GDP-bound states. Studies have shown that Rac1 plays a critical role in a variety of cellular responses, including cell proliferation and survival, gene transcription, adhesion, motility and forming of the actin cytoskeleton^3^. Overexpression of Rac1 has been reported in several types of solid tumors, including breast cancer and pancreatic cancer^4,5^. In hematological malignancies, studies have shown that Rac1 GTPase activated by BCR-ABL represented a novel target in chronic myeloid leukemia. Additionally, Rac1 inhibition delays the development of acute leukemia in a murine model in vivo^6^. However, the role of Rac1 in lymphoma thus far has not been clearly defined.

Rac1-GTP interacts with multiple effectors and activates numerous downstream signaling pathways such as PI3K/Akt, AMPK and ERK pathways^7^. Among them, the Akt signaling is one of the most commonly deregulated oncogenic pathways in MCL. Constitutive activation of the PI3K/Akt/mTOR pathway not only contribute to aggressiveness of MCL, but also crosstalk with other oncogenic pathways such as NF-κB signaling pathway^8,9^. In addition, ERK1/2 pathway is also critical to the proliferation as well as survival of MCL tumor cells through inhibition of BCL-2 family member BCL-XL^10^. These findings suggest that Rac1 is likely to play an important role in the pathogenesis of MCL.

By analyzing the gene expression profiling (GEP) data of 41 MCL cases, we found that Rac1 mRNA is overexpressed in MCL tumor samples (Fig. 1a). We also examined the levels of Rac1 mRNA and protein in a panel of MCL cell lines. The results showed that Rac1 mRNA is overexpressed in four of six MCL cell lines (Jeko-1, Maver-1, Mino and Z138) compared to naive B cells (Fig. 1b), while the Rac1-GTP protein level is markedly increased in all tested MCL cell lines compared to naive B cells (Fig. 1c). It is worth noting that the mRNA expression of Rac1 is not well correlated with its protein level, implying that post-transcriptional or translational regulation plays a part in Rac1 expression in MCL cells.

**Fig. 1:**
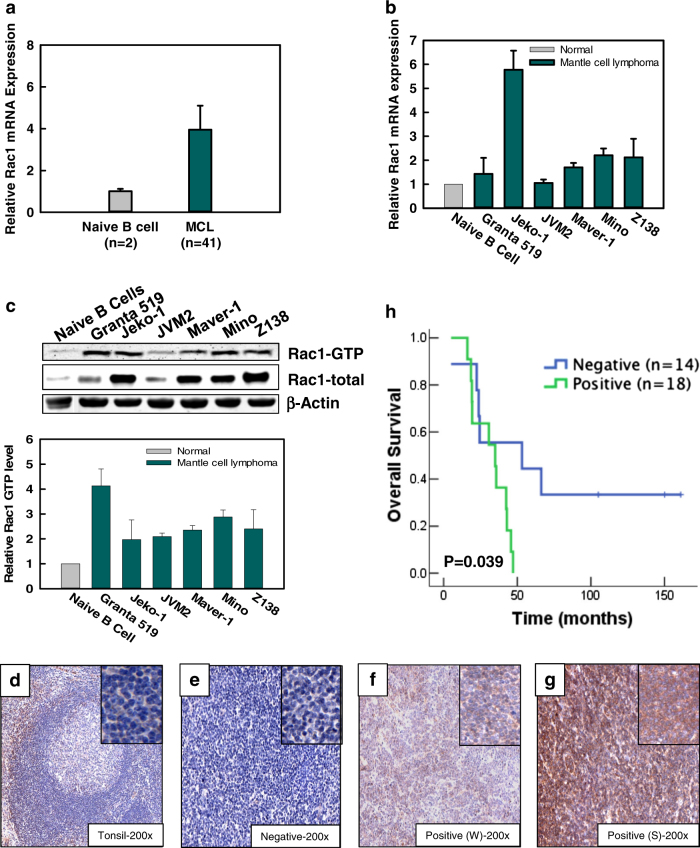
Rac1 is overexpressed in human primary MCL tumors and MCL cell lines. Analysis of Rac1 mRNA levels in (**a**) human primary MCL tissues from the LLMPP database and (**b**) MCL cell lines. **c** Upper panel: Rac1-GTP, Rac1-total and β-actin protein expression in MCL cell lines was analyzed by western blot; lower panel: quantification of Rac1-GTP level by Odyssey CLx system (LI-COR). This calculation was based on the ratio between Rac1-GTP signal and that of Rac1-total. The experiments exhibited in (**b**) and (**c**) were repeated three times, and an average ratio to that of the naive B cells is shown. **d**–**g** Representative images of the immunohistochemistry (IHC) for tonsil (**d**) and MCL lymphoma cases that is negative for Rac1 (**e**), positive for Rac1 (**f**, **g**, W weak Rac1 staining, S strong Rac1 staining). **h** Overall survival of MCL patients in relation to Rac1 protein expression

To confirm the upregulation of Rac1, we performed immunohistochemical (IHC) analysis in 32 MCL cases. In normal lymphoid tissue, mantle zones of follicles were negative for Rac1 (Fig. 1d), whereas 18 cases of MCL (18/32; 56%) showed positive expression for Rac1, with six cases each falling into weak, medium and strong staining groups, respectively (cutoff value 30%) (Supplemental table 1 and Fig. 1f, g). Furthermore, we correlated Rac1 expression with clinical outcome and found that Rac1 positivity was strongly associated with shorter overall survival (OS, *p* = 0.039) (Fig. 1h).

To confirm the significance of Rac1 dysregulation in MCL, the 41 cases were then divided into three groups based on Rac1 mRNA level and correlated with cell proliferation. As shown in supplemental Fig. 1, higher Rac1 level was significantly correlated with higher levels of the proliferation signature, as established in our previous study (*p* < 0.001)^11^. Next, we transduced MCL cell lines Z138 and Mino, which express high levels of both Rac1 and Rac1-GTP, with Rac1-shRNA by inducible retroviral vector. Upon doxycycline (Dox) induction, Rac1 protein level was decreased by 55% and 50% in Z138 and Mino cells, respectively, compared to the vector controls (Fig. 2a). Notably, knockdown of Rac1 decreased cell viability by 43% (*p* < 0.001) and 24% (*p* = 0.002) in Z138 and Mino cells, respectively, after 3 days of continuous culture (Fig. 2b). To confirm the result, we deployed a commonly used selective inhibitor NSC23766^12^ to inhibit Rac1. After 2 h incubation, half maximal effective concentration (EC50) of NSC23766 toward Rac1 inhibition was observed at 25 μM (Supplemental Fig. 2a). Cell viability of Z138 and Mino were substantially inhibited by NSC23766 in a dose-dependent manner within its linear dose range for Rac1 inhibition (Fig. 2c). Notably, naive B cells were barely affected by NSC23766 treatment, even at the 100 μM concentration (Supplemental Fig. 2b). Moreover, we overexpressed Rac1 in Z138, as well as JVM2 cells that express lower level of both Rac1 and Rac1-GTP (Fig. 2d). The result showed that Rac1 overexpression substantially rescued the cells from the inhibitory effect of NSC23766 (Fig. 2e). These results suggest that targeting Rac1 provides a specific toxic effect toward MCL lymphoma cells.

**Fig. 2:**
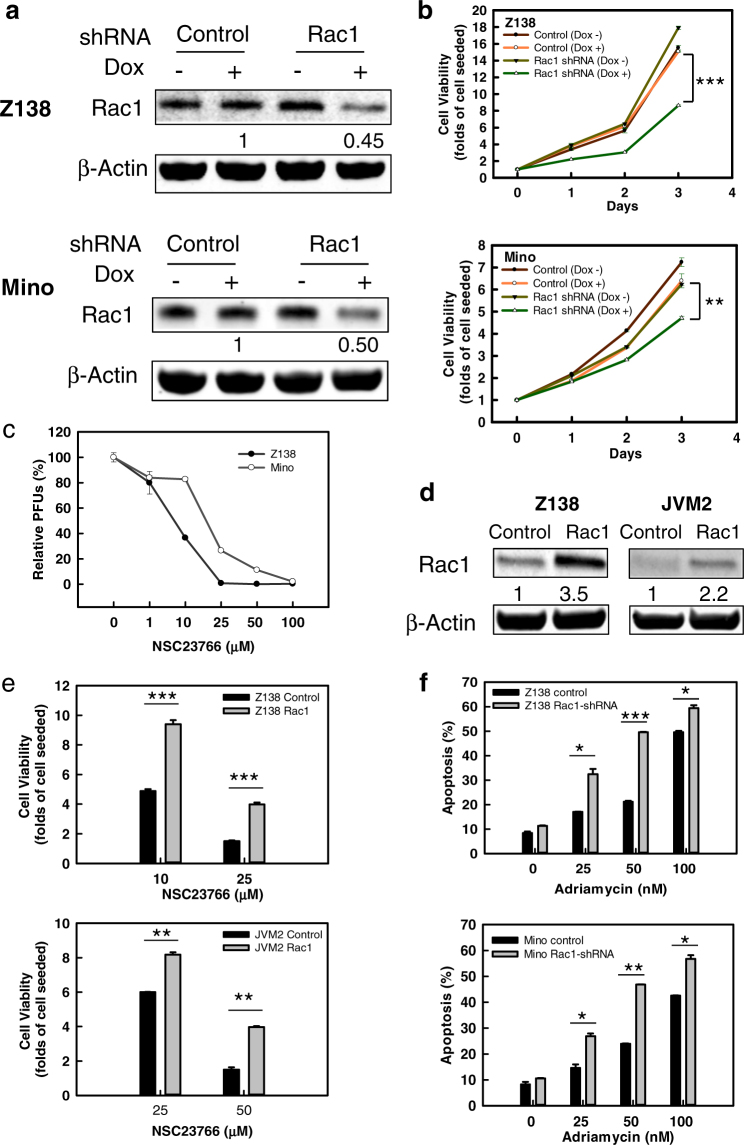
Rac1 promotes cell proliferation and survival in MCL. **a** Rac1 was knocked down by Dox inducible-shRNA in Z138 and Mino MCL cells. The protein level was determined by western blot after 24 h of 1 μg/ml Dox treatment. The relative Rac1 levels in the Dox treated samples were determined by normalization of the Rac1 levels with their respective actin levels. The experiments were repeated three times with similar results and representative blots are shown. **b** Cell viability of Rac1 knockdown MCL cells (Z138 and Mino) and control cells were determined for 3 days by PrestoBlue cell proliferation assay. Define the cells number of each group on day 0 as 1 and the *y*-axis stands for the increased folds of cell numbers on each day compared to the day 0. The experiments were repeated three times in four replicates with similar results obtained. Data shown are the average of three experiments and are presented as mean ± SEM. *P* value stands for the difference between Rac1-shRNA Dox (+) and control Dox (+). **P ≤ 0.01; ***P ≤ 0.001. **c** Z138 and Mino cells were treated with NSC23766 at indicated concentrations for 3 days and assessed for cell viability by PrestoBlue assay. The *y*-axis stands for the percentage of cells number on day 3 compared to the day 0. PFUs: Prestoblue fluorescent units. **d** Exogenous Rac1 was transfected by pcDNA3-EGFP-Rac1-wt in Z138 and JVM2 cells. The protein level was determined by western blot. The relative Rac1 levels in the Rac1-transfected cells were determined by normalization of the Rac1 levels with their respective actin levels. **e** Cell viabilities of Rac1 overexpressed MCL cells (Z138 and JVM2) and vector control cells were determined by PrestoBlue cell proliferation assay at 3 days after treated with NSC23766 at indicated concentration. Define the cells number of each group on day 0 as 1 and the *y*-axis stands for the increased folds of cell numbers on the 3rd day compared to the day 0. The experiments were repeated three times in four replicates with similar results obtained. *P* value stands for the difference between Rac1-transfected group and control group upon NSC23766 treatment. **P ≤ 0.01; ***P ≤ 0.001. **f** Z138 and Mino cells transduced with Rac1-shRNA or control-shRNA were treated with 1 μg/ml of Dox for 24 h, and then treated with increasing concentrations of Adriamycin for 48 h. Cell apoptosis was determined by Annexin V and 7-AAD staining followed by flow cytometry analysis. The experiments were repeated three times with similar results obtained. Data shown are the average percentage of three experiments and are presented as mean ± SEM. *P* value stands for the difference between Rac1-shRNA Dox (+) and control Dox (+). *P ≤ 0.05; **P ≤ 0.01; ***P ≤ 0.001

In view of Adriamycin being a core reagent of R-CHOP regimen, we tested the combination effect of Rac1 depletion with Adriamycin in MCL cells. As shown in Fig. 2f, knockdown of Rac1 in MCL cells had little effect on cell survival. However, when combined with Adriamycin, Rac1 knockdown cells exhibited a nearly 2-fold increase in apoptosis compared to the control cells. Similarly, the addition of NSC23766 to Adriamycin significantly increased the inhibitory effect after 72 h treatment (5.65 ± 0.05 vs. 3.12 ± 0.02 folds increase in apoptosis for Mino, *p* = 0.007; 10.8 ± 0.38 vs. 4.2 ± 0.07 folds increase for Z138, *p* < 0.001), though synergy effect was not clearly demonstrated (Supplemental Figure 3a). We also specifically investigated the cytotoxic effect and found that the combination treatment induced apoptosis ~2.36-fold and 1.68-fold higher in Z138 and Mino cells, respectively, than that of Adriamycin alone treatment (Supplemental Figure 3b). These data suggest that Rac1 inhibition enhances the cytotoxic effect of Adriamycin, further supporting its significance in clinical practice.

To explore the molecular mechanism underlying the functional roles of Rac1 dysregulation, we investigated several oncogenic pathways of MCL upon Rac1 depletion. As shown in Supplemental Figure 4a, Rac1 knockdown substantially diminished the phosphorylation of Akt at both T308 and S473 residues. Consistently, Akt downstream target mTORC1 also exhibited a decreased activity, as shown by diminution in phosphorylation of RPS6 (S235/S236). In addition, phosphorylation of RelA/p65 (S536) and ERK1/2 (T202/T204), indicative of activation of these proteins, were also decreased upon Rac1 knockdown. Similar effect was observed when MCL cells treated with NSC23766 (Supplemental Figure 4b). These results suggest that Rac1 overexpression plays important role in the hyper-activation of multiple oncogenic pathways in MCL.

Gene rearrangement involving *Cyclin D1* is the hallmark of MCL. However, it has been demonstrated that Cyclin D1 overexpression alone is insufficient to induce the onset of MCL^13^, raising the importance of additional mechanisms in MCL lymphomagenesis. Consistently, several core oncogenic pathways including Akt and NF-κB signaling have been found to be dysregulated without correlated genomic aberrations in MCL, which implies an interactive activation of pathway networks in the cancerous state. Here we demonstrated that Rac1 is directly associated with the activation of several prosurvival oncogenic pathways in MCL, suggesting that it locates at the central node of pathway network. However, the mechanism underlying Rac1 overexpression in MCL remains unclear. Previous studies have demonstrated that endogenous Rac1 is activated by B-cell antigen receptor (BCR) signaling and is required for the subsequent activation of BCR downstream signal transduction^14^. Considering the constitutive activation of the BCR signaling in MCL^15^ and its wide connections with other oncogenic pathways, it is likely that Rac1 is an important integrator of activating signals in MCL cells. Future studies are expected to elucidate the Rac1 regulation network and its significance in signaling integration.

We found that the inhibition of Rac1 only brought cytostatic effect. This is in agreement with the notion that defects of the pro-apoptotic machinery, such as BCL-2 and MCL1 overexpression, render MCL tumor cells resistance to chemotherapy-induce apoptosis. Notably, inhibition of Rac1 significantly enhanced the cytotoxic effect induced by Adriamycin, potentiating the combination of Rac1 inhibition with R-CHOP in clinical practice. Although in vivo studies are needed to demonstrate the effectiveness, our findings provide evidence supporting a proof-of-concept to target Rac1 as a novel therapeutic strategy for the treatment of MCL.

## Electronic supplementary material

supplemental information
