# Supplementary material for: Rac1 is a novel therapeutic target in mantle cell lymphoma
Source: Blood Cancer J. 2018 Feb 12;8(2):17. doi: 10.1038/s41408-018-0052-0 (PMC5809391; doi:10.1038/s41408-018-0052-0)
Supplement: Supplementary file 1 — supplemental information [file 41408_2018_52_MOESM1_ESM.pdf]

SUPPLEMENTAL MATERIALS AND METHODS

Cell culture and chemical reagents

MCL cell lines (Jeko-1, JVM2, Mino, Maver-1 and Z138 cells) were cultured in RPMI 1640 supplemented with 10% fetal bovine serum (FBS; Invitrogen). Granta-519 was cultured in DMEM culture medium with 10% FBS. All those cells came from ATCC and were authenticated and tested without mycoplasma contamination. Naïve B cells were isolated from peripheral blood mononuclear cells using a naïve B cell isolation kit (Miltenyl Biotec, CA). Rac1 inhibitor NSC23766 was purchased from Tocris Biosciences (Ellisville, MO).

Immunohistochemistry

Tissue from 32 MCL cases and 2 Tonsils were obtained from the University of Nebraska Medical Center, embedded in paraffin and arranged into tissue micro-arrays (TMAs). TMAs were deparaffinized in xylene followed by rehydration through graded ethanol. Endogenous peroxidase was blocked using peroxidase quenching solution (SuperPicture 3rd Gen IHC Detection Kit, Life Technology) for 10 min. The slides were then boiled in citrate buffer (pH 6.0, Dako) for 1 hour, allowed to cool and rinsed with PBS. The sections were incubated overnight at 4 ℃ with primary antibody (Rac1 1:100, #23A8 from EMD MILLIPORE) in SignalStain antibody diluent (Cell Signaling Technology). Slides were then washed with PBS and incubated with HRP polymer conjugate (SuperPicture 3rd Gen IHC Detection Kit) for 1 h at room temperature, followed by PBS wash and then stained with DAB reagent. Photographs were taken using Roche Ventana Scan HT. All clinical samples and features of the patients were retrieved from the clinical database of Department of Pathology and Microbiology at UNMC. This study was approved by the institutional review board of UNMC.

Gene expression profiling

Previously, the Leukemia and Lymphoma Molecular Profiling Project (LLMPP) conducted a comprehensive gene expression profiling (GEP) of MCL specimens. As a result, a set of proliferation genes (proliferation signature) were identified representing a biological integrator of oncogenic events associated with cell proliferation, which allowed the definition of prognostic subgroups that differ in median survival by more than 5 years. In our study, GEP of 41 molecularly defined MCL cases from the LLMPP were analyzed for Rac1 expression. Moreover, patients were divided into three groups according to their Rac1 mRNA expression levels (high, median and low Rac1 groups, see Table 1), then gene expression-based proliferation signatures between Rac1 high and low groups was analyzed.

Antibodies and immunoblotting

Cell lysates were separated and immunoblotted by using Bolt® Bis-Tris system (Life technology, Carlsbad, CA). Imaging and quantitative analyses were performed by Odyssey CLX system (LI-COR Biosciences, Lincoln, NE). The antibodies for immunoblots are as follows: Akt (#4685), p-Akt (#4060), p-Akt (#13038), p-RPS6 (#4858), S6RP(#2217), 4E-BP1 (#9644), p-4E-BP1 (#2855), p-ERK1/2 (#4370), ERK1/2 (#4695), p-P65 (#3033) and P65 (#8242) were all from Cell Signaling Technology (Danvers, MA); β-actin (#SC-47778) was from Santa Cruz Biotechnology (Dallas, TX); Rac1 (#23A8) was from EMD MILLIPORE (Billerica, Massachusetts).

Quantitative reverse transcription PCR (qRT-PCR)

RNA was isolated using The Quick-RNA™ MiniPrep (Plus) kit (ZYMO Research). The SuperScript®III First-Strand Synthesis System (Invitrogen) was used for cDNA

synthesis. Real-time PCR was performed using DyNAmo HS SYBR Green qPCR kit (Thermo Scientific Inc.) with CFX connect (Bio-Rad, Hercules, CA) real-time thermocycler. The ΔCT method was used to calculate the relative mRNA expression level by normalizing with the expression of housekeeping gene *RPL13A*. QRT-PCR primers used in this study are as follows: *Rac1* forward: *CTGATGCAGGCCATCAAGT; Rac1 reverse: TCTCCAGGAAATGCATTGGT. RPL13A* forward: *AGATGGCGGAGGTGCAG*; *RPL13A* reverse: *GGCCCAGCAGTACCTGTTTA.* .

Rac1 activity assay

A glutathione-S-transferase (GST)-PAK1 (PAK-CRIB domain) fusion protein, containing the Rac1 binding region from human PAK1B, was used to determine Rac1 activity as described. Mino cells were pretreated with serum starvation (1% FBS containing medium) for 24h, then treated with different concentrations of NSC23766 for 2 hours. This was followed by adding epidermal growth factor (EGF) (Sigma-Aldrich) at 100 ng/mL for 15 min, re-suspension in lysis buffer (50 mM Tris-HCL (pH 7.4), 100 mM NaCL, 2 mM $MgCL_2$ and 1 mM benzamidine; 1% NP-40; 10% glycerol; 1 µg/mL leupeptin, pepstatin, and aprotinin) and centrifugation at 1500 rpm for 5 min at 4°C. For negative control, supernatant protein were pretreated with 10mM EDTA (Boston BioProducts, Inc., Ashland, MA) and 1 mM GDP (20-177, EMD Millipore) for 15 min at 30°C with rotation. Equal amounts of supernatant protein (including negative control tube) were incubated with the GST-PAK1 fusion protein bound to glutathione-coupled Sepharose beads at 4°C for 1h with rotation to capture GTP-bound Rac1. Beads were washed 3 times with lysis buffer and eluted in Laemmli buffer (60 mM Tris (pH 6.80), 2% sodium dodecyl sulfate, 10% glycerin, 0.1% bromophenol blue). SDS-PAGE was performed on samples using a 4-20% gel and bound Rac1 analyzed by immunoblotting.

Rac1 shRNA knockdown in MCL cell lines

For the inducible shRNA expression, miR-E based shRNA was cloned into retroviral vector p-Retro-x (Clontech, CA). Virus was packaged by co-transfecting GP2-293T cells with constructed vectors and pCL-ampho. After 48h of transfection, virus-containing medium was collected and precipitated with PEG at 4℃ overnight. Targeted cells were then transduced with concentrated lentivirus with TransDux (System Biosciences, CA) and incubated for 48h followed by selection with puromycin. ShRNA expression was initiated by doxycycline (Dox) at 1 µg/mL and the knockdown efficiency was determined by Western blot after 24h of induction. In all, three shRNAs were tested and the one with the highest knockdown efficiency was chosen for subsequent functional studies. The Rac1-shRNA sequences are shRNA1: *gggcatttaattcatcttta*, and shRNA2: *cgacactgtcacttgaccaa*.

Analysis of viability and apoptosis

Cell proliferation and viability were determined by PrestoBlue cell proliferation/viability assay kit (Invitrogen, CA) following the manufacturer's instructions. Fluorescence intensity was measured using Plate reader Infinite® 200PRO (Tecan, Switzerland) at 560 nm for excitation and 590 nm for emissions. Apoptosis was determined by Annexin V-FITC and propidium iodide (PI), or Annexin V-PE and 7-AAD staining (BD Pharmingen) according to the manufacturer's instructions followed by quantification using a FACS Calibur flow cytometer (BD Biosciences).

Statistical analyses

For correlation and survival analyses, patients were divided into two groups according to Rac1 immunohistochemistry (IHC) staining (cutoff: 30%). Pearson's w2-test

was used to analyze correlations between Rac1 status and overall survival (OS) or event free survival (EFS). Survival was estimated using the Kaplan–Meier method, and the comparison between study groups was performed with the log-rank test. The end-point EFS was calculated as time from diagnosis to the first documented recurrence.

For in vitro experiments, group comparisons were evaluated using the two-tailed, unpaired *t*-test (equal variance). All statistical analyses were performed using SPSS 16.0 and are shown as means ± SD (standard deviation). P-values <0.05 was considered significant

Supplemental Table 1.
Rac1 protein expression in MCL tissues

IHC	Percentage	Case number
Positive (≥30%)	>75%	6
	55%-75%	6
	30%-55%	6
Negative (<30%)	<30%	14

SUPPLEMENTAL FIGURE LEGENDS

Supplemental Figure 1: 41 MCL cases with gene expression profiling data were divided into three groups (n=14 in each group) based on the Rac1 mRNA expression. Heatmap illustrating the proliferation signature in Rac1-low and Rac1–high mRNA expression cases.

Supplemental Figure 2: Inhibition of Rac1 by NSC23766 inhibits cell proliferation in MCL cells. (**a**) Mino cells were treated with NSC23766 at the indicated concentrations for 2h. Cell lysates were subjected to Rac1 activity assay as described in *Materials and Methods*. Relative Rac1 activity in each sample is calculated by normalizing the Rac1-GTP level with total Rac1 level. (**b**) Naïve B cells were treated with 100 μM NSC23766 for the indicated number of days and cell viability was determined by PrestoBlue assay. Define cells number of each group on day 0 as 1 and the Y-axis stands the increased folds of cells number on each day compared to the day 0. The experiments were repeated three times in four replicates with similar results obtained, and an average PresoBlue fluorescence unit is shown. All data shown are the average of three experiments and are presented as mean ± SEM.

Supplementary Figure 3. Inhibition of Rac1 by NSC23766 enhances the cytotoxic effect of Adriamycin. (**a**) Z138 and Mino cells were treated with NSC23766 and/or Adriamycin for the indicated time and cell viability was determined by PrestoBlue assay. The experiments were repeated three times with similar results obtained. P value stands for the difference between combined treatments and Adriamycin treated group. **, $P \leq 0.01$; ***, $P \leq 0.001$. (**b**) Z138 and Mino cells were treated with NSC23766 and/or Adriamycin for 48h and apoptosis determined by Annexin V and PI staining, as described in *Materials and Methods*. The experiments were repeated three times with similar results obtained. Data shown are the average of three experiments and are presented as mean ± SEM. *, $P \leq 0.05$; **, $P \leq 0.01$.

Supplementary Figure 4. (a) Rac1-shRNA expressing and control cells were examined for phosphorylation of Akt, RPS6, NF-κB (p65), ERK1/2 and β-actin by immunoblotting after 24h Dox induction. Ctr, control. (b) Z138 and Mino cells were treated with 50 μM NSC23766 for the indicated time (0, 1, 4, 8, 24h) and followed by Western blot analyses for the indicated protein phosphorylation and total levels.

Sup Fig. 1

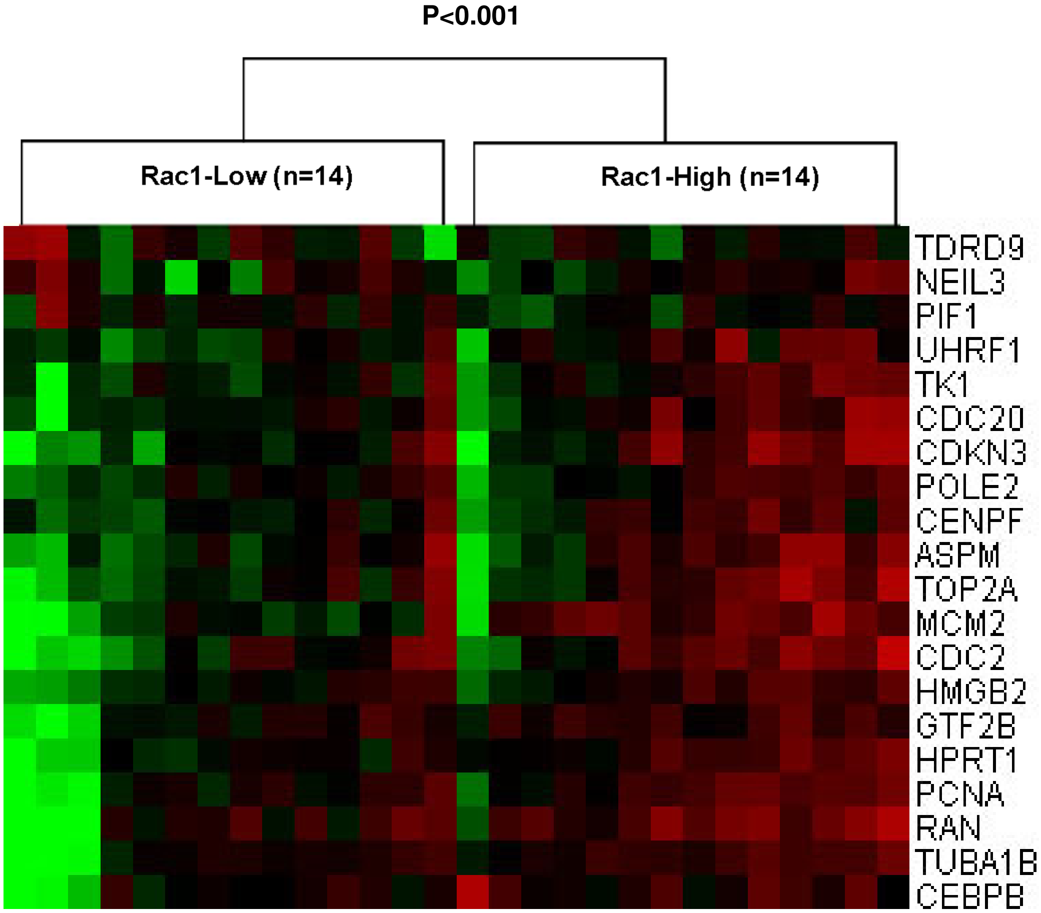

P<0.001
Rac1-Low (n=14)
Rac1-High (n=14)
TDRD9
NEIL3
PIF1
UHRF1
TK1
CDC20
CDKN3
POLE2
CENPF
ASPM
TOP2A
MCM2
CDC2
HMGB2
GTF2B
HPRT1
PCNA
RAN
TUBA1B
CEBPB

Sup Fig. 2

a

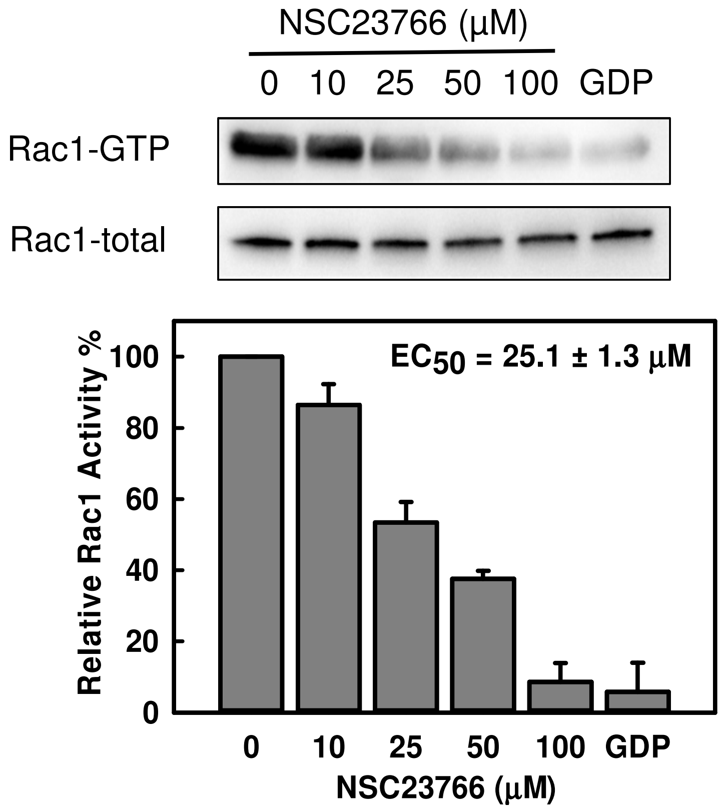
NSC23766 (μM)
0 10 25 50 100 GDP
Rac1-GTP
Rac1-total
EC50 = 25.1 ± 1.3 μM
Relative Rac1 Activity %
100
80
60
40
20
0
0 10 25 50 100 GDP
NSC23766 (μM)

b

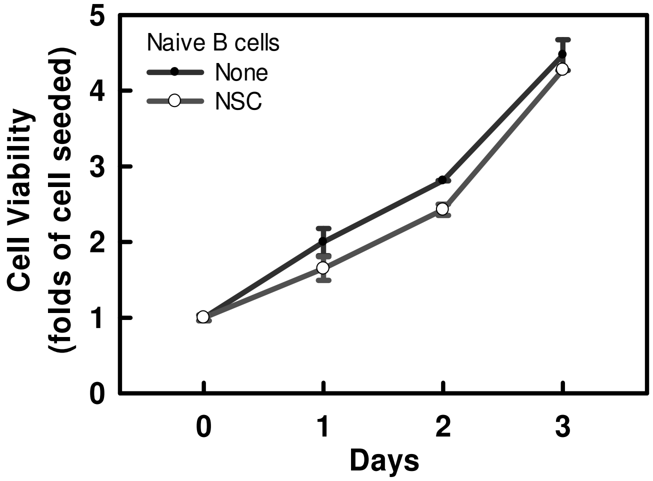
Naive B cells
None
NSC
Cell Viability
(folds of cell seeded)
5
4
3
2
1
0
0 1 2 3
Days

Sup Fig. 3

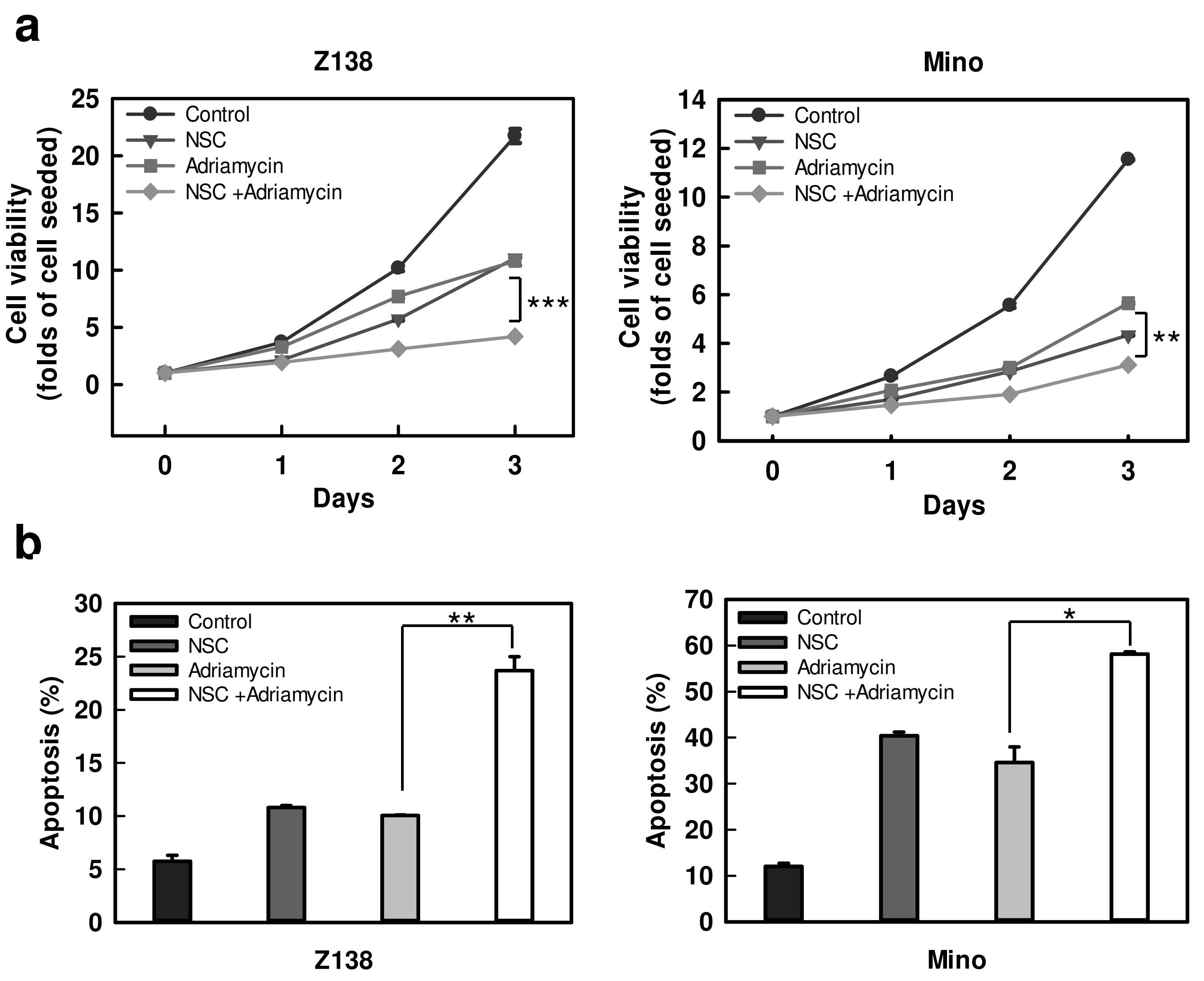
a
Z138
Mino
Cell viability (folds of cell seeded)
Control
NSC
Adriamycin
NSC +Adriamycin
***
**
Days
0
1
2
3
b
Apoptosis (%)
Control
NSC
Adriamycin
NSC +Adriamycin
**
*
Z138
Mino

Sup Fig. 4

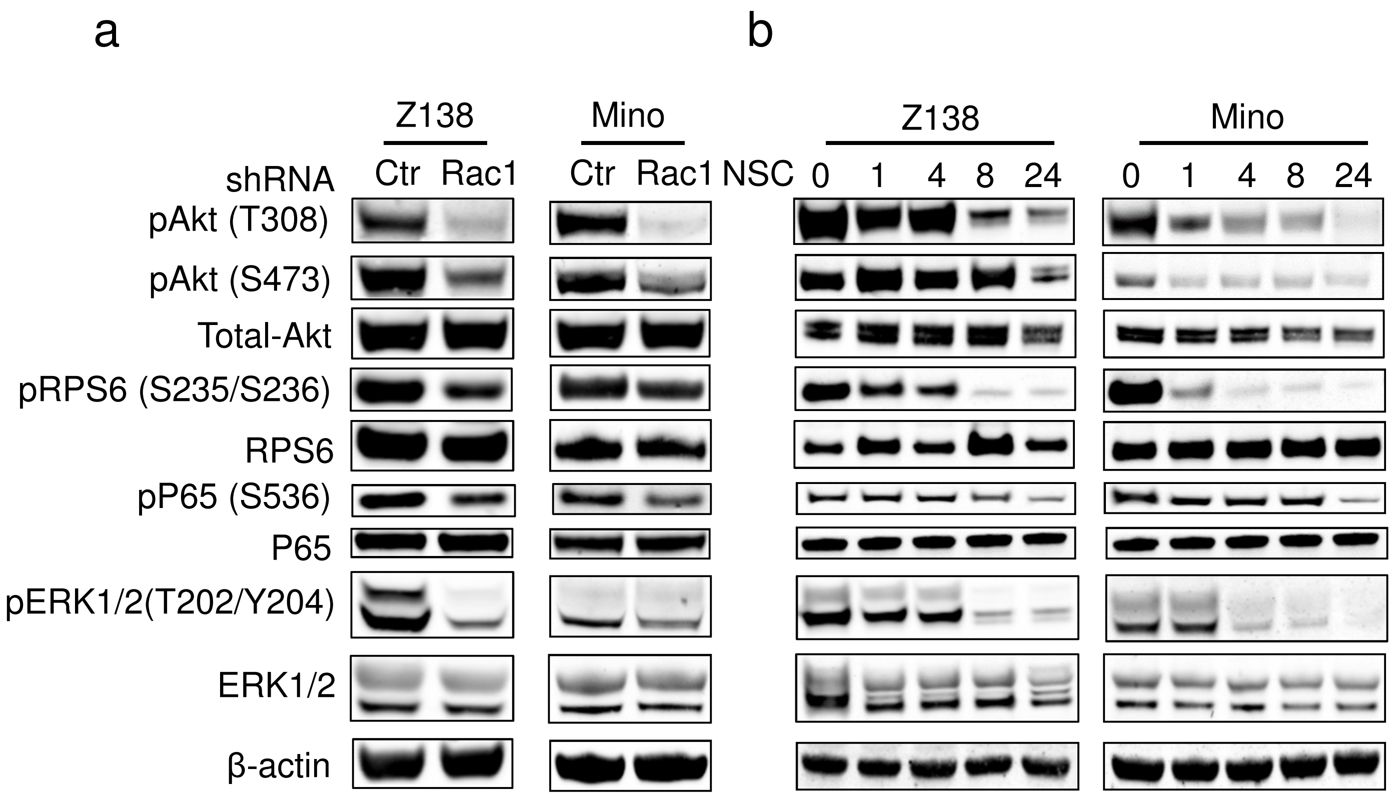
a
b
Z138
Mino
Z138
Mino
shRNA
Ctr
Rac1
Ctr
Rac1
NSC
0
1
4
8
24
0
1
4
8
24
pAkt (T308)
pAkt (S473)
Total-Akt
pRPS6 (S235/S236)
RPS6
pP65 (S536)
P65
pERK1/2(T202/Y204)
ERK1/2
β-actin
